# Defining the care delivery value chain and mapping the patient journey in rheumatoid arthritis

**DOI:** 10.1007/s00296-022-05215-z

**Published:** 2022-09-23

**Authors:** Fiona Koster, Deirisa Lopes Barreto, Sandhya C. Nair, Marc R. Kok, Angelique E. A. M. Weel-Koenders

**Affiliations:** 1 Department of Rheumatology and Clinical Immunology, Maasstad Hospital , Rotterdam, The Netherlands; 2 Erasmus School of Health Policy and Management, Erasmus University Rotterdam, Rotterdam, The Netherlands; 3 Real World Evidence and Analytics, Global Commercial and Strategy Organization, Janssen Pharmaceutica NV, Beerse, Belgium

**Keywords:** Rheumatoid arthritis, Health care delivery, Patient participation, Patient care episodes

## Abstract

Rheumatoid Arthritis (RA) is a chronic disease that impacts patients’ quality of life. Sophisticated organization of care delivery drives quality improvement. Therefore, the study objective was establishing a validated process map of the care cycle for RA patients. Hence, increasing transparency and optimizing care delivery and identifying areas of improvement. To map the RA care cycle, the care delivery value chain (CDVC) approach was used as framework to document activities and resources systematically. A mixed method study was conducted where quantitative data on activities were collected from health records and unstructured interviews with medical staff were held. Consequently, the process map was separately validated in a consensus meeting with a delegation of the medical staff and patient advisory board. At the start of the care cycle, the focus is predominantly on defining the treat-to-target strategy and examining disease activity. Towards the monitoring phase, tapering medication and managing the disease through patient-reported outcome measures are becoming increasingly important. Although patient’s functioning, quality of care and patient’s evaluation of received care are monitored, reflection of CDVC and engaging patients in the evaluation process resulted in improvement actions on outcome and process level. Mapping the RA care cycle following a systematic approach, provides insight and transparency in delivered activities, involved resources and the engagement of patients and caregivers at multiple levels, contributing to a system facilitating value-based care delivery. The CDVC framework and applied methodology is recommended in other conditions. Future research will focus at assigning outcomes and costs to activities and evaluating interventions to explore patient value.

**Supplementary Information:**

The online version contains supplementary material available at 10.1007/s00296-022-05215-z.

## Introduction

Rheumatoid Arthritis (RA), the most common form of rheumatic disease, is an autoimmune illness affecting joints and connective tissues [1]. Over the past decades, significant improvements in the treatment of RA have been accomplished. However, patients still experience impairments in their daily life [2]. One way to measure the impairments from the patient perspective is through patient-reported outcome measures (PROMs), mainly used for research purposes [3].

The shift towards a more patient-centered healthcare system enhances the role of the patient in daily practice regarding treatment decisions and subsequent outcomes [4]. Therefore, PROMs are considered to be central components within Value-Based Health Care (VBHC), as PROMs reflect outcomes from the patient perspective, aiming to improve the perceived value [5, 6]. Patient involvement within the VBHC strategy also induces the alignment of patients with respect to the process of integral healthcare delivery and therefore on the denominator of Porters’ value equation, which defines patient value as health outcomes divided by costs [4].

Mapping patient pathways or so-called journeys in a systematic manner, provides insight in the value added by as well as the (in)efficiencies related to the provision of care of each activity, may reduce practice variation and on the other hand promote personalized care at the right place [7]. Several methods and frameworks to design patient journeys are available [7]. However, most methods lack a direct link to the relevant outcomes, e.g. clinical and/or PROMs, as a measure of the quality of care besides the healthcare costs involved. Within VBHC, the Care Delivery Value Chain (CDVC) was developed as a framework to facilitate the construction of patient journeys, encompassing patient relevant activities for a given medical condition [6]. Furthermore, the patient journey can be exploited in co-creation with patients to redesign the care cycle to improve the quality of the care provided as well as identifying the proper time horizon to analyze (patient reported) outcomes [7, 8]. Also, identified inefficiencies can be resolved to enhance value creation. The CDVC approach will be exploited to assess the organization of RA care at Maasstad hospital The objective of this study is to define the CDVC and to establish a detailed process map of the care delivered within the standardized pathway criteria, i.e. RA patients following the regular patient journey. Areas of value creation will be identified based on the structured and practical mapping of the RA care cycle in collaboration with patients and the medical treatment team.

## Methods

### Study design and data collection

A mixed method research design was followed to assess the CDVC and map the activities concerning the patient journey. Quantitative data concerning the CDVC were collected from electronic health records. The Dutch Healthcare Authority requires medical personnel to register the performed care activities per patient and therefore the electronic health records contain detailed information on the healthcare procedures carried out within the patient journey.

### Study population and setting

#### Quantitative

The prospective (open) cohort research was conducted in a real-life cohort of RA patients at the rheumatology department of Maasstad Hospital a top-clinical research hospital in Rotterdam, the Netherlands, from 2014 onwards. The inclusion criterion for the study was a RA diagnosis determined by a rheumatologist. Currently, over 3,100 diagnosed patients yearly medical care for RA in Maasstad hospital. Gender, disease duration and age at diagnosis were analyzed in the study population. StataSE version 15 was used for the descriptive statistics.

#### Qualitative

The qualitative data including time frames and division of labor were obtained from in-depth interviews with the staff members of the rheumatology department. Over 40 people work in the department, of which 12 are rheumatologist. The department functions as a training institute for rheumatologists and research is a high priority. The catchment area of the hospital is the fourth largest in the country.

The study started with a process map for the rheumatology department dating from 2014, which was updated by conducting interviews with the rheumatology medical team (supplementary file 1). The inclusion criterion of the cohort applied, was that one staff member per employer group was allowed to give unstructured feedback on the process map. Revisions were incorporated and the updated process map was sent to the treatment team of rheumatologists. A delegation of the rheumatologists validated the full care cycle by means of a single focus group. The researcher presented the patient journey including all activities, resources and timeframes in a chronological manner. An unstructured methodology was chosen, allowing medical staff members to raise any question regarding the RA care cycle.

#### Co-creation with patients

In 2016 a patient advisory board was initiated at the Maasstad hospital and in 2022 the board comprises of approximately 70 inflammatory rheumatic disease (IRD) patients of which around half suffer from RA. The purpose of the panel is multifold as Maasstad hospital strives to achieve active patient engagement and participation. Depending on the purpose of a gathering, events and meetings are held to inform, consult, ask for advice, co-create, co-decide with or stimulate self-management by IRD patients. For study purposes the RA care cycle was presented to the patient advisory board to evaluate and co-create the delivery of RA care from the patient perspective.

### Outcomes

#### Care delivery value chain and process map

The CDVC was established by applying the method of Porter et al. to map and evaluate the process of care delivery [6]. The CDVC describes the main activities within the patient journey as well as the process flow and organization of the care delivery cycle. In the CDVC, inter- and intrapersonal communication between medical personnel are not incorporated. A detailed process map, including all activities, of the patient journey was constructed with the CDVC phases serving as a base. As part of the Dutch healthcare system, registration of the activities is required to properly reimburse the provided care [9]. The Dutch reimbursement system comprises over four thousand reimbursement codes, allowing for a detailed analysis of the individual care activities [9]. The process map gives an overview of the trajectory patients must follow regarding the activities as part of their RA treatment. Non-reimbursed activities such as registration of patients at the reception desk, were identified via interviews. The clustering in the CDVC was based on the output of the interviews with both the staff and the patients.

#### Improvement and optimization of the RA care cycle

Through the mapping of the CDVC we aimed to identify value improvements opportunities or areas concerning the optimization of the care delivery. The focus of the identification of improvement actions is mainly organizing care in alignment with the demands of patients suffering from a chronic disease.

##### Patient engagement

To examine the level of patient participation in the care delivery process, the framework of Carman et al. was applied [8]. In that framework three levels of engagement are distinguished: direct care, organizational design and governance and policy making [8]. The latter level of patient engagement is, however, not applicable for this study. For each level of engagement, the extent of patient participation is either on basis of consultation, involvement or partnership and shared leadership, i.e. the continuum of engagement [8].

### Data visualization

Microsoft Visio (version 2016) was used to visualize the care cycle of RA patients [10]. Flowcharts consist of swimming lanes, delineating the total cycle in sub processes. Swimming lanes are either horizontal or vertical arranged. With respect to the RA patient journey, horizontal swimming lanes are used to distinguish the staff members and resources essential for the individual process steps. Staff members are denoted on the vertical axis in the margin of the swimming lanes. The patient journey starts in the upper-left corner of the flowchart. The representation of the flowchart symbols are described in the results section.

## Results

### Population characteristics

As of 2021, the Maasstad hospital RA population comprised of 3141 patients of which 71.5% female. The mean age at diagnosis is 57.7 (SD = 15.0) and on average the disease duration is 7.8 years (SD = 4.6).

### Care delivery value chain

**Fig. 1:**
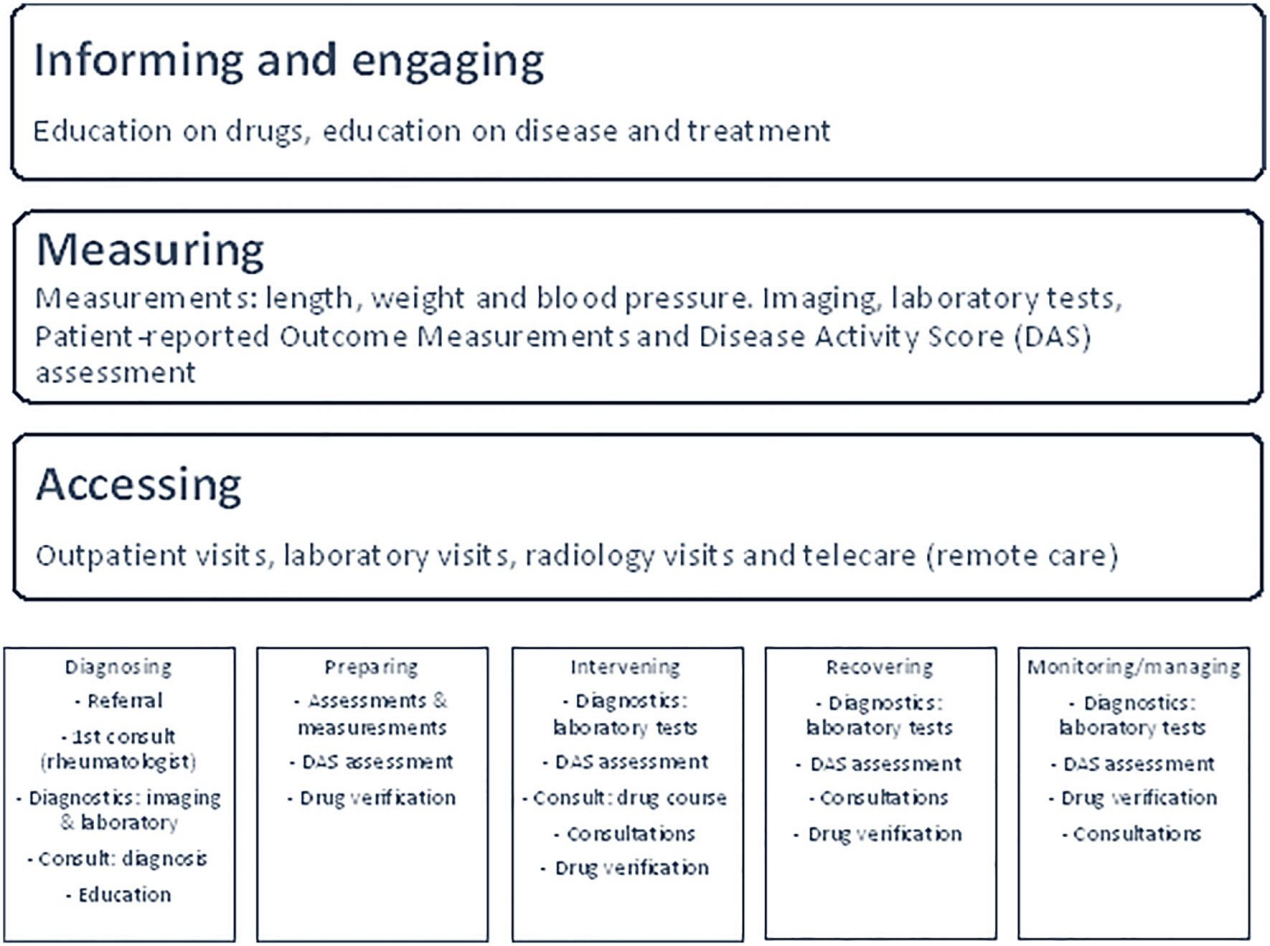
Care delivery value chain of rheumatoid arthritis patients

In Fig. 1 the CDVC for RA patients is displayed. Per element of the CDVC, the main activities with respect to the treatment of RA patients are summarized. The CDVC distinguishes eight different elements concerning the mapping of a care cycle, of which the bottom five describe the phases relevant for the actual process mapping. Informing and engaging, measuring and assessing occurs throughout the phases of the CDVC. In the following sections, these phases and the corresponding activities are described in detail for the RA care cycle.

### Informing and engaging, measuring and accessing

#### Informing and engaging

Education concerning the disease (e.g. shared-decision making) and the pharmacological treatment, as part of the informing and engaging process, is mainly provided in the first half year after the diagnosis (Table 1). After this initial phase, the treatment team provides education on request or when needed, for example when patients switch between drugs or taper medication intake. Apart from education provided by staff members, patients can actively engage by virtue of the ReumaWeb application, which is a self-management tool designed to provide remote coaching consisting of information and exercises on how to alleviate RA-related complaints [11].

**Table 1:** Informing and engaging, measuring and accessing within Rheumatoid Arthritis care cycle

Phase	# Of activities	Informing and engaging	Measuring	Accessing
Diagnosing	11	Disease education	Screening disease activity, i.e. blood tests and x-rays	GP practice Outpatient rheumatology department Laboratory Radiology
Preparing	4	Education, i.e. drugs and non-drug Drug information	Disease activity measurement (DAS28) Baseline measures (length, weight blood pressure)	Outpatient rheumatology department Hospital pharmacy
Intervening	12	*PROMs education and information* Education, i.e. drug side effects	Screening disease activity, i.e. blood tests	Laboratory Outpatient rheumatology department Hospital pharmacy
Recovering (from symptoms)	12	Upon request or when needed *ReumaWeb app®*	Screening disease activity, i.e. blood tests Disease activity measurement (DAS28)	Laboratory Outpatient rheumatology department Hospital pharmacy
(Life-long) monitoring and managing	7	Upon request: education, i.e. drugs *ReumaWeb app®*	Disease activity measurement, i.e. DAS28 Patient Reported Outcome Measures (DQRA, FACIT, EQ-5D, HAQ-DI, POP-66, RAID, WPAI)*	Laboratory Outpatient rheumatology department Remote consultations Hospital pharmacy
Total	46			

*DQRA Dutch quality registry rheumatoid arthritis, *FACIT* Functional assessment of chronic illness therapy fatigue scale, *HAQ-DI* Health assessment questionnaire disability index, *RAID* Rheumatoid arthritis impact of disease, *WPAI* Work productivity and activity impairment

Italics as part of research

#### Measuring

At the beginning of the care cycle, the patient’s medical condition is assessed by measuring the disease activity. Towards the monitoring phase PROMs are becoming increasingly important. In addition to the usual clinical examinations, PROMs are gradually integrated as part of the daily clinical practice to represent the outcomes that matters most to RA patients and help to determine treatment goals. For this purpose, the ICHOM standard set for inflammatory diseases was implemented measuring various health domains [12]. Prior to implementing the PROMs at the department, four panel meetings with patients from the patient advisory board were held to discuss and evaluate the PROMs questionnaires and the tool visualizing the results [13]. In addition to RA-related health outcomes, social participation of the patients is also observed by examining productivity at work and mental well-being. Patient’s evaluation of the care delivered is integrated in the PROMs amid the DQRA, a quality register for care facilities treating Rheumatoid Arthritis in the Netherlands. As the DQRA measures patients' perceptions of their care delivery experience, the DQRA is considered as a PREM (Patient Reported Experience Measure).

#### Accessing

Sites of care delivery are most often the various departments in the hospital as displayed in Table 1.

### Patient journey RA

The detailed process map considering the RA population, i.e. patients suffering solely from RA, is presented in the supplementary materials (supplementary file 2). On average, patients visit the rheumatologist and the doctors assistant six times and the physician assistant/nurse practitioner three times in the first year. Visits to the rheumatology nurse total three and counter employees perform 15 activities within the patient journey. Drugs are picked up at the hospital pharmacy during (six of) the onsite visits. Diagnostics, i.e. imaging and blood testing, is conducted eight times.

### Diagnosing

The diagnostic phase of the RA care cycle contains 11 distinctive steps, involving four different healthcare professionals. The degree of variation is largest in the diagnosing phase with respect to the staff members patients consult and the succession of onsite appointments. The focus of this phase is to properly diagnose patients within three weeks, but as timely as possible. Based on the diagnosis, patient-tailored education is provided.

### Preparing

The preparing phase consists of five steps. Since this phase commences immediately after the diagnosis, patients are not compelled to visit the outpatient department solely for the preparing phase. Informing patients on the nature of their illness is of importance as it may affect the treatment responsiveness positively as a result of therapy compliance. Therefore, the target of the preparing phase is to increase patient activation through supporting and engaging patients in the treatment by informing.

### Intervening

The intervention phase follows the preparation phase. A total of 11 activities were distinguished in the intervening phase; the process is divided in two parts, taking place with an interval of approximately six weeks. The corresponding steps in the parts are similar, however the consultation with the rheumatologist is replaced by a consultation with the physician assistant or the nurse practitioner. The focus of the initial intervention phase in RA is obtaining a suitable treatment setting for patients, i.e. treat-to-target. Treat-to-target is setting a clinical target such as low disease activity or remission, choosing the treatment through shared-decision making and accomplishing the clinical target [14]. Assessment of the determined target is conducted, in consultation with the patient, via for example the disease activity score, the primary clinical outcome measure advise by international guidelines [15].

### Recovering (from symptoms)

In general, the recovery (from symptoms) phase begins three months subsequent to the intervention and consists of 11 steps. Activities in the recovery phase are comparable to the activities in the intervention phase. However, the focus is on achieving the (agreed) clinical target. And, if possible, tapering of the drugs are pursued without causing a flare-up of the disease. Since RA is a chronic disease and full recovery is unattainable, the designation is adjusted to the recovery or stabilization from symptoms. During recovery, the disease activity of the patient is expected to decrease as a result of effective treatment.

### (Life-long) monitoring and managing

Monitoring and managing starts on average after 48 weeks of treatment and continues thereafter. In case of inflammation of the joints or a high disease activity, due to the nature of the illness, patients will deviate from the time span illustrated in the patient journey. With respect to the conventional care process, the steps for patients are in line with the recovery phase. The frequency of consultations in the monitoring phase varies between the different staff members and is dependent on whether a patient experiences flare episodes. After one year the DAS assessment and blood drawing takes place approximately every 12 to 24 weeks, depending on the level of disease activity of a patient. In total, monitoring and managing regular care counts seven successive steps.

### Validation process map

Once the detailed process map was charted, the patient journey was presented and distributed amongst the members of the rheumatology patient advisory board. Patients were encouraged to provide comments on the patient journey either during the meeting or via e-mail. At the board meeting, the patients confirmed the outlined steps and therefore the validation did not lead to significant adjustments within the process map. Furthermore, no e-mails were received concerning comments to the patient journey. With respect to the validation amongst the medical staff of the rheumatology department, a few comments were made concerning the stated time frames and were discussed during the presentation of the patient journey. Therefore, one adjustment was made with respect to the time frame within the diagnostics stage.

### Improvement and optimization of the care cycle

#### Patient engagement

To improve the care delivery, patients are stimulated to actively participate in the evaluation of the patient journey. Regarding the first level, i.e. direct care, patient’s engagement is at the higher end of the engagement continuum. This is due to the fact that treatment is based on patient preferences through shared-decision making and PROMs assessment. The intermediate level, involving patients, applies to organizational design and governance (second level). Perspectives of the patients in the patient advisory board are considered in the design and evaluation of the care process as described below.

#### Process level

After analyzing the patient journey by means of the CDVC, several improvement areas were mentioned during the interviews and meetings with patient partners and caregivers. Pertaining to the accessing phase, it was noticed that the care sites are primarily the hospital departments. With the recent development of shifting healthcare beyond the hospital walls and the COVID-19 pandemic, a telemonitoring track was initiated by Maasstad hospital to expand the access to healthcare and to reduce the burden on patients. Despite the fact that patients can have electronic consultations, it is still necessary to visit the hospital for blood drawing. By facilitating drawing blood at home, the full monitoring phase will be shifted from the hospital site to the home of patients, reducing transportation costs and time burden on patients. Patients participating in the patient panel indicated their preference of blood drawing at home as opposed to the hospital. The suggested telemonitoring track was also discussed and approved by the staff of the rheumatology department. In the near future, in addition to the performed e-consultations, patients will be able to carry out blood drawing at home.

### Outcome level

An outcome level improvement was identified to support the performance of DAS assessments at home as part of the telemonitoring track. The standard DAS assessment will be substituted or complemented by a patient-reported DAS assessment in the telecare process. As a result, patients are able to fill out the DAS assessment remotely and are not required to visit the hospital. Another outcome improvement action was related to the measuring phase of the CDVC. To measure PRO’s in a consistent manner, the improvement action focuses on a frequent measurement. Therefore, patients are requested to fill out the PROMs every six months in line with the ICHOM recommendations. A baseline measure will be performed in the preparation phase. Thereafter, patients will be requested to complete the PROMs semi-annually.

#### Structure level

Concerning the improvements with respect to the organization of the RA healthcare delivery, another improvement area was identified. Applying PROMs as guidance to arrange the recurrence of appointments in the monitoring and managing phase. Thereby enhancing patient involvement and patient value since care delivery is customized to individual patients. The timing and organization concerning the education given by rheumatology nurses is also investigated. In the former situation, education was solely provided after the diagnosis in the preparing phase. As a result of a focus group with 16 patients, a second consultation with the rheumatology nurse is added to the patient journey to gain more knowledge at the follow-up in the intervening phase.

## Discussion

The results of the study demonstrated that by applying a mixed method design the CDVC is a useful method to structure and gain insight in the real-life care delivery cycle of RA patients. Despite the clustering in the CDVC process steps, many of the performed procedures in the steps are similar. Yet, the focus of the different process steps in the care cycle vary. The focus shifts, from predominantly a treat-to-target strategy and examining disease activity at the start of the cycle, towards improvement on tapering medication and managing the disease on a more personalized basis through PROMs. Although the patient’s-maintained functioning, quality of care and the patient’s evaluation of the received care are monitored, reflection of the CDVC and patient journey resulted in several improvements on outcome and process level.

The CDVC is considered as the basis for the integration of VBHC in healthcare delivery. By applying this methodology, we have gained knowledge concerning the arsenal of the delivered chronic care services, ultimately leading to personalized value of RA treatment (personalized outcomes related to personalized journey cost). A perspective facilitating to complement and synergize the classical focus of evidence based medicine and towards improving value by reorganization of the care delivery on the meso and macro level, where besides clinical evidence also evidence on patient report outcomes and healthcare cost are included [16], 17. Ultimately, the goal is to provide personalized care on a micro level by engaging patients through consulting, involvement and evaluation of the care delivery process [8]. Moreover, the creation of the process map and mixed methodology of the RA patient journey allows for uniform analyzes and can therefore be considered as a blueprint for other (chronic) illnesses.

The self-reported DAS, one of the improvement actions, is a next step in the treatment of RA patients remotely. However, a possible challenge could be the level of reproducibility of the self-reported DAS to a clinical reported DAS. A systematic review conducted by Rampes et al. demonstrated that both the total joint count (TJC) and small joint count (SJC) were reliable when performed by patients [18]. In a similar study, the CDVC was created for HIV/AIDS in Togo, enabling the identification of quality improvements with respect to the chronic care delivery for pediatric HIV and AIDS [19]. However, a distinction was the fact that the care delivery consists of many more facets in comparison to the RA care cycle. Moreover, in this study, the CVDC was utilized to construct a detailed process map and to determine the level of patient engagement in the care delivery process. To our knowledge, combining the development of the CDVC and patient journey with the framework of Carman et al. to investigate the level of patient engagement in RA care delivery has not been carried out before. The latter is of importance from the VBHC perspective, as the input of patients and patient engagement should be enhanced.

In prior research the RA patient journey was developed by conducting interviews with patients and combining the results of the interviews with process mapping [20]. The focus of the study by Oliver et al. was predominantly on depicting patient experiences concerning the care delivery. A validated graphical representation of the process was however lacking. Moreover, implications for improvements were mainly aimed the access to rheumatology care. Hence, in this study an illustration of the patient journey is given on basis of the CDVC and the improvement actions are not confined to specific phases of the care cycle. Another methodology, as mentioned by van Weert and Hazelzet (2020) [21], is presenting the patient journey through so-called “metro mapping”, in which the patient journey is depicted as a metro line [22].To date the majority of metro mapping is performed within elective (oncology) disciplines rather than chronic care and the metro lines method restricts the application of loops, returning to a previous activity, within the care cycle. Furthermore, the metro mapping of care cycle is conducted by educated service designers, making it a less accessible method compared with the CDVC, which can be performed by anyone of the organization [22].

An additional strength is related to the engagement of healthcare providers in the process of mapping, improving the efficiency of the delivery of care and identifying practice variation as processes were discussed and, in the end, formalized. Moreover, the established CDVC and detailed process map were presented and discussed with the patient panel. Defining improvement actions and the patient care implementation of telecare in the CDVC to increase patient value are additional strengths of the research. A last strength is attributed to the financial organization of the Dutch healthcare system where care activities are broadly registered and information with respect to care activities within patient journey are easily accessible. Therefore, activities and costs can also be easily matched.

A limitation of the study is the fact that the patient journey was limited to RA patients treated in the hospital silo (secondary care). The integral process, i.e., primary care and tertiary care, could not be included in the mapping of the patient journey due to the pillarization of the Dutch healthcare system. However, the majority of the outcome and procedures is carried out at secondary care institutions and therefore, insight in this part of the patient journey is of great relevance. A second limitation of the study concerns the process steps described in the CDVC. Allocating activities to the phases of the CDVC is to a certain extent subjective and can therefore be interpreted differently, potentially complicating the CDVC benchmark. However, the guidelines with respect to the care of RA are nationally established, Moreover, as the CDVC was validated by the medical staff and the patient advisory board the potential bias is limited. The validation of the care cycle was limited to an internal validation, which can be considered a final limitation as it reduces the generalization of the results. a next step is to externally validate the CVDC and process map within other hospitals in- and outside of the Netherlands.

### Conclusion and future perspectives

The aim of the study was to define the patient journey for the standardized care delivery in single-morbid RA patients. However, in the remaining, around one-fifth of the patients, the pathway may deviate due to patient’s characteristics e.g. multimorbidities, preferences and unforeseen circumstances. Therefore, further investigation is required to address challenges such as the occurrence of multimorbid and complex patient populations in the care cycle. As a next step, allocation of healthcare costs to the activities within the CDVC, will provide insight into the (integral) healthcare costs associated with different patient journeys. Obtaining and allocating the related costs and time of the RA CDVC allows for a sophisticated analysis of the care cycle, contributing to the transition to a value-based healthcare system. In the end, the goal is to evaluate outcomes in relation to costs on a patient level. In conclusion, establishing the CDVC and process map for RA provided detailed information regarding the patient journey of RA patients in an effective and value attaining manner. The study provided transparency in and standardized the various processes, activities and involved staff members in the delivery of RA care. As a result, improvement areas were easily identified, and the implementation of patient telecare were facilitated.

## Supplementary Information

Below is the link to the electronic supplementary material.

Supplementary file1 (PNG 230 KB)

Supplementary file2 (JPG 399 KB)
