# Supplementary figures and images for: Defining the care delivery value chain and mapping the patient journey in rheumatoid arthritis
Source: Rheumatol Int. 2022 Sep 23;43(4):743–50. doi: 10.1007/s00296-022-05215-z (PMC9510493; doi:10.1007/s00296-022-05215-z)

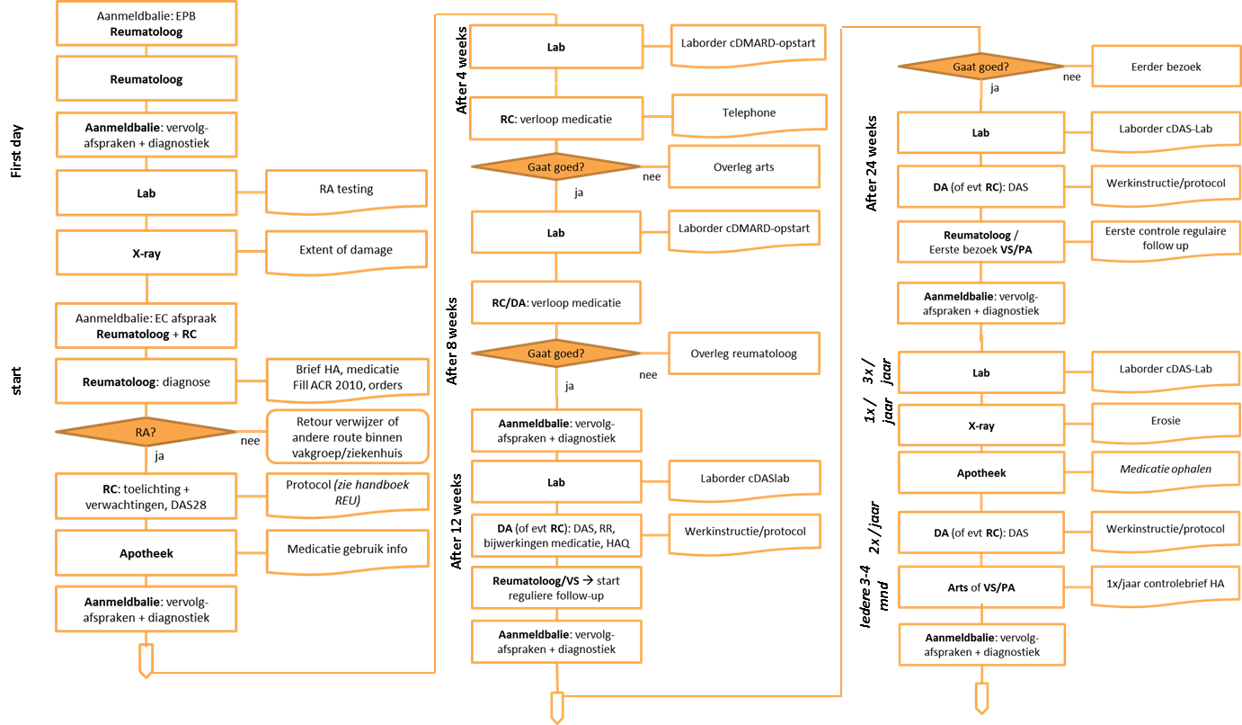

Supplement: Supplementary file 1 — Supplementary file1 (PNG 230 KB) [file 296_2022_5215_MOESM1_ESM.png]

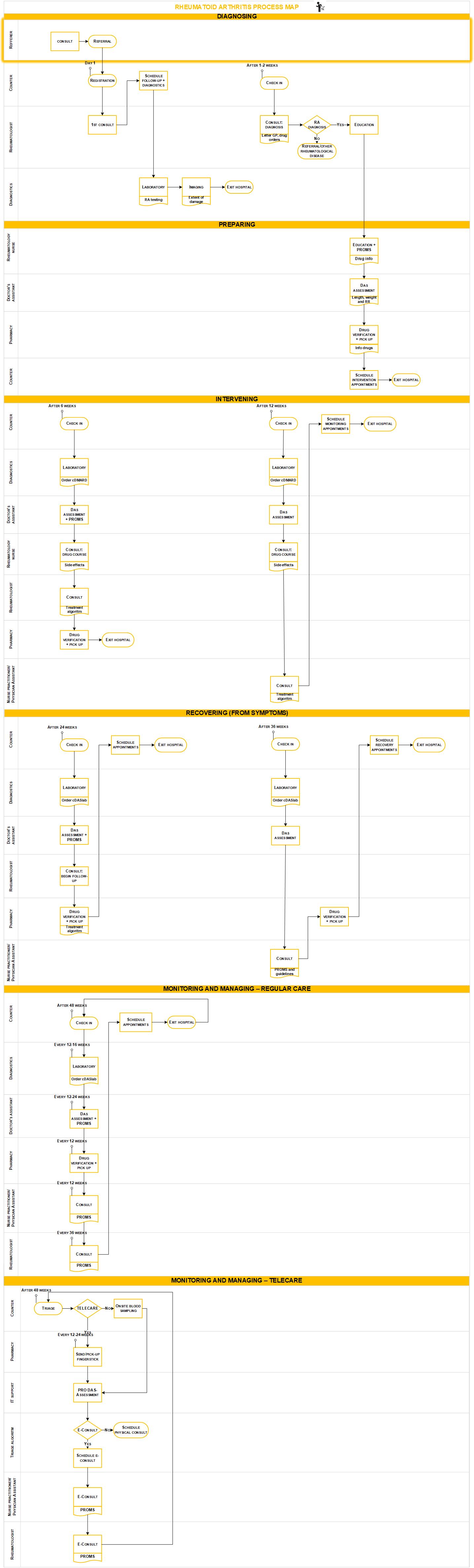

Supplement: Supplementary file 2 — Supplementary file2 (JPG 399 KB) [file 296_2022_5215_MOESM2_ESM.jpg]
